# Calcium Signaling as an Emerging Integrator of Manganese Homeostasis in *Arabidopsis*: From Molecular Mechanisms to Adaptive Strategies

**DOI:** 10.3390/plants15091396

**Published:** 2026-05-02

**Authors:** Xiaoyun Zhang, Baochen Zhang, Ye Wang, Lijuan Zeng, Zhixuan Wen, Ming Lei, Li Li

**Affiliations:** 1 School of Modern Industry for Selenium Science and Engineering, National R&D Center for Se-Rich Agricultural Products Processing Technology, Wuhan Polytechnic University, Wuhan 430023, China; 13125026916@163.com (X.Z.); 19863923995@163.com (B.Z.); wyzb20002023@163.com (Y.W.); 15123418958@163.com (L.Z.); wzx18986011556@163.com (Z.W.); 2 Guangxi Key Laboratory of Medicinal Resources Protection and Genetic Improvement/National Center for Traditional Chinese Medicine Inheritance and Innovation/Guangxi Engineering Research Center of Traditional Chinese Medicine Resource Intelligent Creation, Guangxi Botanical Garden of Medicinal Plants, Nanning 530023, China

**Keywords:** manganese homeostasis, calcium signaling, transporter regulation, CNGC channel, IRT1, deficiency, toxicity, adaptive strategies

## Abstract

Manganese (Mn) is essential for plants, but its fluctuating soil availability—deficiency in alkaline soils and toxicity in acidic soils—challenges crop productivity. Breakthroughs in *Arabidopsis* have uncovered Ca^2+^ signaling as a key integrator of Mn status. This review synthesizes these discoveries into an emerging *Arabidopsis*-centered framework. Under Mn deficiency, sustained Ca^2+^ oscillations activate CPK21/23, which phosphorylate the importer NRAMP1 at Thr498 to enhance Mn uptake. Under Mn excess, a rapid Ca^2+^ transient triggers a multi-layered cascade: CPK4/5/6/11 activates MTP8 (Ser31/32) for vacuolar sequestration, while CBL2/3–CIPK3/9/26 sequentially suppresses MTP8 (Ser35, peak 24 h) and MTP11 (Ser194/201, peak 36 h)—a multi-tiered “brake” system. Concurrently, CBL1/9–CIPK23 induces NRAMP1 endocytosis (Ser20/22) to limit Mn uptake. The IRT1 transporter directly binds cytoplasmic Mn^2+^ and triggers its own degradation via CIPK23, thereby converging with Ca^2+^ signaling. The BRI1–CNGC12 module generates Mn-induced Ca^2+^ signals. By organizing current knowledge into a hierarchical framework, this review provides a working model for future research and outlines translational opportunities for engineering Mn-resilient crops.

## 1. Introduction

Manganese (Mn) is an indispensable micronutrient for plants, serving as a cofactor for enzymes central to photosynthesis (the oxygen-evolving complex of photosystem II), mitochondrial superoxide dismutation (MnSOD), and cell wall biosynthesis (glycosyltransferases) [1,2]. These reactions occur in different cellular compartments, including the chloroplasts, mitochondria, and Golgi apparatus—necessitating precise spatial and temporal control of Mn distribution [3]. The importance of Mn for crop productivity is underscored by the severe consequences of its deficiency. Typical deficiency symptoms include interveinal chlorosis of young leaves, stunted growth, delayed maturity, and reduced grain yield [4]. In cereals, Mn deficiency causes specific disorders such as ‘grey speck’ in oats, ‘marsh spot’ in peas, and ‘split leaf’ in barley, leading to substantial economic losses [5,6]. In soybeans, Mn deficiency reduces nodulation and nitrogen fixation, further compromising productivity [7]. Soil Mn bioavailability is highly pH-dependent: in alkaline and calcareous soils, Mn is oxidized to insoluble Mn oxides, causing deficiency, whereas in acidic soils, Mn^2+^ is abundantly available, frequently leading to toxicity [8,9]. Acidic soils now account for more than 30% of global arable land, making Mn toxicity the second most limiting factor for plant growth on such soils after aluminum toxicity [10,11].

Over the past two decades, research has catalogued an extensive repertoire of plant adaptations to Mn stress. Under deficiency, plants remodel their root architecture, upregulate high-affinity transporters, and remobilize internal Mn stores [12,13,14]. Under excess conditions, they activate antioxidant systems, sequester Mn into vacuoles and cell walls, chelate Mn with organic acids, and reprogram metabolism [15,16,17]. Despite this progress, a unifying framework explaining how plants integrate deficiency and excess signals has been missing.

Recent studies have filled this gap by revealing an unexpected role for Ca^2+^ signaling in Mn homeostasis. Mn excess triggers a rapid Ca^2+^ transient that activates CPK4/5/6/11, which phosphorylates the vacuolar Mn transporter MTP8 at Ser31/32 to enhance sequestration [18]. Mn deficiency, by contrast, induces sustained Ca^2+^ oscillations that activate CPK21/23, which phosphorylate the plasma membrane importer NRAMP1 at Thr498 to increase Mn uptake [19]. A second regulatory layer involves CBL2/3–CIPK3/9/26 complexes, which phosphorylate MTP8 at Ser35 to suppress its activity as a brake [20]. Very recently, the same CBL2/3–CIPK3/9/26 module was shown to phosphorylate the Golgi-localized Mn efflux transporter MTP11 at Ser194/201, triggering its degradation and Golgi retention, thereby reducing Mn exocytosis under prolonged stress [21]. Additionally, CBL1/9–CIPK23 phosphorylates NRAMP1 at Ser20/22 under Mn excess, inducing its endocytosis [22]. A Ca^2+^-independent parallel pathway involves ROP6 GTPase, which directly binds MTP8 to enhance its vacuolar localization [23]. Upstream of these responses, the brassinosteroid receptor BRI1 phosphorylates the Ca^2+^ channel CNGC12 at Ser22 to generate Mn-induced Ca^2+^ signals [24].

Parallel to these Ca^2+^-dependent mechanisms, the iron-regulated transporter IRT1 exemplifies direct metal sensing. IRT1 transports Mn^2+^ as a secondary substrate under iron deficiency and uses a histidine-rich loop to monitor cytoplasmic Mn^2+^ levels, triggering its own degradation via CIPK23 [25,26]. This pathway converges with Ca^2+^ signaling at CIPK23.

These discoveries position Ca^2+^ signaling as a key integrator of Mn status in *Arabidopsis*. Here, ‘key integrator’ refers to a signaling node that receives multiple Mn-related inputs, decodes them via distinct kinase modules (CPKs and CBL–CIPKs), and coordinates the activity of multiple Mn transporters (NRAMP1, MTP8, and MTP11) as well as downstream adaptive responses. This review synthesizes the emerging Ca^2+^-centric framework through three interconnected sections: (i) the Ca^2+^ signaling machinery, which senses and transduces Mn status; (ii) downstream adaptive strategies; and (iii) species-specific variations and translational opportunities. Importantly, the direct molecular evidence for Ca^2+^-dependent regulation of Mn transporters is currently limited to *Arabidopsis thaliana*, specifically for NRAMP1, MTP8, and very recently MTP11 [21]. Whether the same regulatory modules operate in crops, hyperaccumulators, or other plant species remains to be tested. We organize current knowledge into a provisional hierarchical model to guide future research, while explicitly distinguishing established mechanisms from hypothetical connections.

## 2. Physiological Imperative for Mn Homeostasis

### 2.1. Essential Functions and Subcellular Demand

Mn^2+^ fulfills non-redundant roles in several organelles. In chloroplasts, the Mn_4_CaO_5_ cluster of the oxygen-evolving complex catalyzes water-splitting [1]. Mn also stabilizes thylakoid membranes and activates ATP synthase, thereby contributing to photosynthetic efficiency [27]. In mitochondria, Mn is an essential cofactor of MnSOD, which is the primary scavenger of superoxide radicals [28]. In the Golgi apparatus, Mn^2+^ activates glycosyltransferases that synthesize cell wall polysaccharides and glycoproteins [29,30]. Mn also serves as a cofactor for pyruvate carboxylase and phosphoenolpyruvate carboxylase in the tricarboxylic acid cycle [2]. These spatially distributed demands require precise Mn delivery and buffering mechanisms.

### 2.2. Consequences of Imbalance

Mn deficiency primarily impairs photosystem II, reducing electron transport and causing interveinal chlorosis [4,31]. In Scots pine seedlings, electron transport efficiency is reduced by 38% under Mn deficiency, directly accounting for the diminished photosynthetic performance [32]. Lignin biosynthesis is also compromised, weakening the cell wall integrity and disease resistance [5]. Nitrogen assimilation declines due to the reduced activity of Mn-dependent nitrate reductase and glutamine synthetase [7]. Conversely, excess Mn generates reactive oxygen species (ROS), damages chloroplast ultrastructure, and inhibits chlorophyll synthesis [17,33]. The narrow window between deficiency and toxicity—tissue concentrations of 20–40 mg kg^−1^DW are sufficient for most crops, while above 500 mg kg^−1^ often causes damage—underscores the need for tight homeostatic control [15]. How plants achieve this precise regulation has been elucidated through recent studies on Ca^2+^ signaling, as detailed below.

## 3. Ca^2+^ Signaling: An Emerging Regulatory Hub

### 3.1. Mn-Induced Ca^2+^ Signatures and Their Molecular Generators

In *Arabidopsis* roots, Mn status generates distinct Ca^2+^ signatures. Mn deficiency (0 µM MnSO_4_) induces sustained oscillations (period ~30 min) in the root elongation zone: a region termed the ‘low-manganese sensing niche’ (LMnSN). By contrast, Mn excess (1.5 mM MnSO_4_) triggers a rapid transient (lasting 2–3 min) at the root meristem/elongation zone border, termed the ‘high-manganese sensing niche’ (HMnSN) [18,19].

The molecular generators of these signals are beginning to be elucidated. Under Mn excess, the cyclic nucleotide-gated channel CNGC12, together with CNGC11, is required to generate Ca^2+^ elevations in the HMnSN, and the brassinosteroid receptor BRI1 directly phosphorylates CNGC12 at Ser22 to activate channel function [24]. Downstream of these Ca^2+^ signatures, distinct kinase modules are engaged. Under Mn deficiency, CPK21/23 decodes the oscillatory signals to phosphorylate the high-affinity Mn importer NRAMP1 [19], whereas under Mn excess, CPK4/5/6/11 transduces the Ca^2+^ transient to activate the tonoplast Mn transporter MTP8 [18].

High-resolution imaging reveals the spatial dynamics of these Ca^2+^ signals. Each oscillation travels inward from the epidermis to the cortex and then to the stele; the signal decline follows the reverse path. The stele may amplify and propagate the signal, as longitudinal expansion occurs only after Ca^2+^ peaks in this tissue [19]. Both sensing niches share the common feature of spatial confinement to specific root zones, yet they generate kinetically distinct Ca^2+^ patterns that are optimally suited to their physiological roles: the transient signal under excess enables rapid detoxification, while the sustained oscillations under deficiency allow continuous monitoring of diminishing Mn availability and sustained upregulation of Mn uptake. The precise coincidence of the LMnSN with the NRAMP1 expression domain [19] and the HMnSN with MTP8 functional zones underscores the spatial coupling between signal generation and effector action in Mn homeostasis.

### 3.2. Direct Evidence: Ca^2+^-Regulated Mn Transporters

#### 3.2.1. Deficiency Response: CPK21/23–NRAMP1 Axis

**Figure 1:**
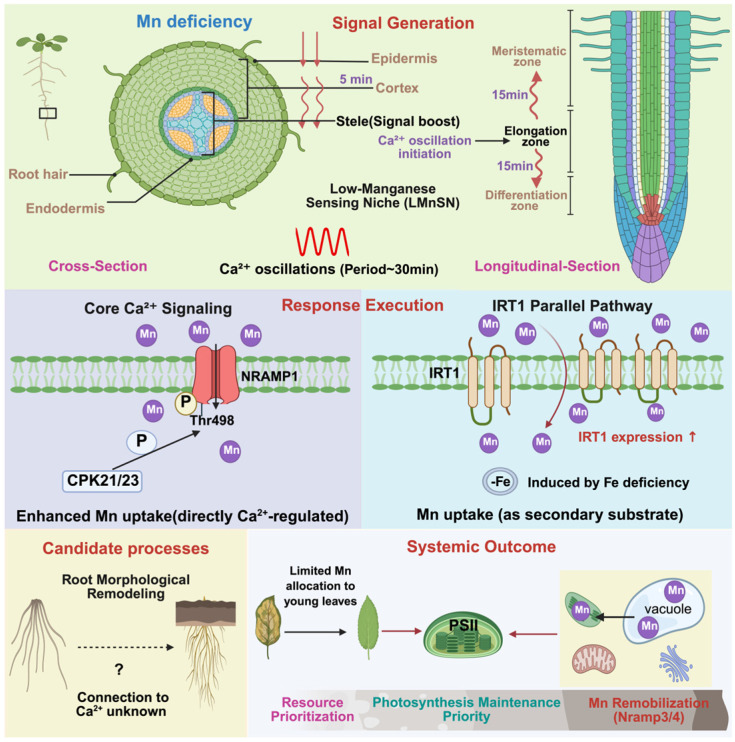
Spatial Coordination of Mn Deficiency Responses in *Arabidopsis*. (**Top panel**) Signal generation. Mn deficiency induces sustained Ca^2+^ oscillations in the root elongation zone (LMnSN). (**Middle panel**) Deficiency response. CPK21/23 phosphorylates NRAMP1 at Thr498 (purple, Ca^2+^-dependent), enhancing Mn uptake (see Section 3.2.1). IRT1 (blue, Ca^2+^-independent) is upregulated by Fe deficiency and contributes to Mn uptake as a secondary substrate (see Section 3.4). (**Bottom panel**) Candidate processes (dashed box, ‘?’ indicates hypothetical connections). (**Left**) Root remodeling may assist Mn acquisition. (**Right**) Systemic effects are manifested as prioritized Mn allocation to young leaves and vacuolar Mn remobilization via NRAMP3/4 (see Section 4.2). Abbreviations: CPK21/23, calcium-dependent protein kinases; NRAMP1, natural resistance-associated macrophage protein 1; IRT1, iron-regulated transporter 1; PSII, photosystem II. Color coding: green, signal generation; purple, Ca^2+^-dependent; blue, Ca^2+^-independent; yellow, candidate processes; gray, systemic outcome; Solid arrows, established connections; dashed arrows, hypothetical.

In *Arabidopsis* roots under Mn deficiency, CPK21/23 phosphorylates NRAMP1 at Thr498, enhancing its Mn transport activity without altering its subcellular localization or stability. Functional analyses using phosphomimetic (T498D) and phospho-dead (T498A) variants have established that Thr498 phosphorylation is both necessary and sufficient for the enhanced Mn transport activity of NRAMP1 under deficiency. The T498D variant confers increased Mn uptake in yeast and fully rescues the *nramp1* mutant phenotype in plants, whereas T498A only partially restores function, indicating that phosphorylation at this site is a major but not exclusive determinant of NRAMP1 activity. Importantly, phosphorylation does not alter NRAMP1 subcellular localization or protein stability, pointing to regulation at the level of intrinsic transport activity rather than trafficking or turnover [19]. The *cpk21 cpk23* double mutant lacks the Mn deficiency-induced increase in NRAMP1 activity and exhibits heightened sensitivity to low Mn (0 µM MnSO_4_), confirming the physiological relevance of this regulatory module (Figure 1, middle panel).

#### 3.2.2. Excess Response I: CPK4/5/6/11–MTP8 Activation

**Figure 2:**
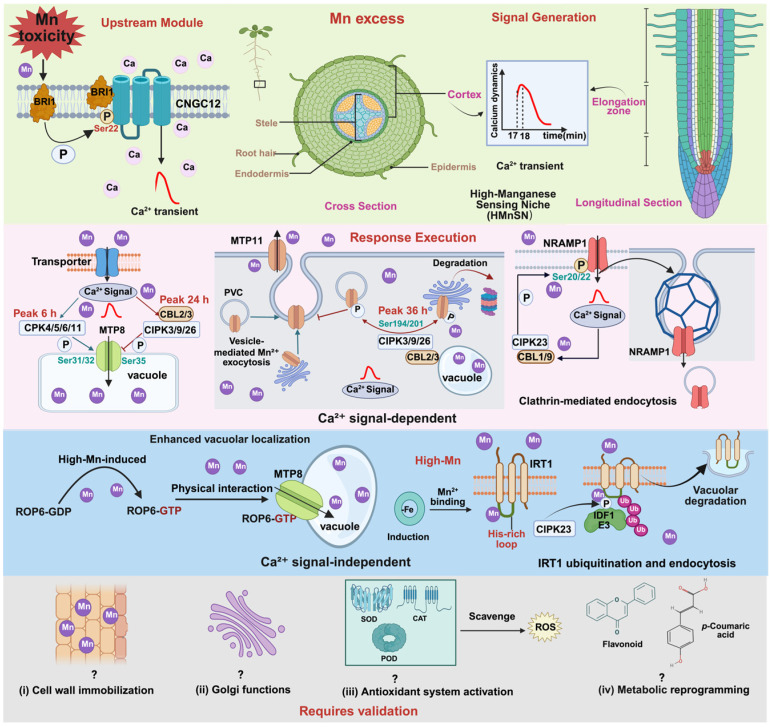
Hierarchical and Temporal Regulation of Mn Excess Responses in *Arabidopsis.* Top layer (green): Signal generation. Mn excess triggers a Ca^2+^ transient in the HMnSN via the BRI1–CNGC12 module (green). Middle layer—pink: Directly Ca^2+^-regulated. MTP8 activation: CPK4/5/6/11 → MTP8 Ser31/32 (peak 6 h) → vacuolar Mn sequestration (see Section 3.2.2). MTP8 suppression: CBL2/3–CIPK3/9/26 → MTP8 Ser35 (peak 24 h) → inhibits vacuolar uptake (see Section 3.2.3). MTP11 suppression: CBL2/3CIPK3/9/26 → MTP11 Ser194/201 (peak 36 h) → degradation & Golgi retention → inhibits Mn exocytosis (see Section 3.2.5). NRAMP1 endocytosis: CBL1/9–CIPK23 → NRAMP1 Ser20/22 → clathrin-mediated endocytosis (see Section 3.2.4). Middle layer—blue: Ca^2+^-independent pathway. ROP6 binds MTP8 (see Section 3.3); IRT1 metal-sensing degradation (see Section 3.4). Bottom layer (gray): Indirect/candidate processes (connections hypothetical, ‘?’). Includes cell wall immobilization, Golgi functions, antioxidant system activation, and metabolic reprogramming (see Section 4.3). Abbreviations: BRI1, brassinosteroid insensitive 1; CNGC12, cyclic nucleotide-gated channel 12; CPK, calcium-dependent protein kinase; CBL, calcineurin B-like protein; CIPK, CBL-interacting protein kinase; MTP8/11, metal tolerance protein 8/11; NRAMP1, natural resistance-associated macrophage protein 1; ROP6, Rho-related GTPase of plants 6; PVC, prevacuolar compartment. Color coding: red, Ca^2+^-dependent; blue, Ca^2+^-independent; green, upstream sensor; gray, indirect/candidate. Solid arrows, established; dashed arrows, hypothetical.

Under Mn excess, CPK4/5/6/11 phosphorylates MTP8 at Ser31/32, activating vacuolar Mn sequestration. Functional redundancy among these CPKs is evident from genetic complementation: phospho-dead MTP8^S31/32A^ fails to rescue the Mn-sensitive mtp8 mutant, whereas the phosphomimetic variant MTP8^S31/32D^ confers enhanced tolerance. Only the cpk4/5/6/11 quadruple mutant, not single *cpk* mutants, phenocopies the *mtp8* mutant [18]. This activation phase represents an emergency detoxification response that quickly sequesters excess cytoplasmic Mn into vacuoles. Notably, CPK-mediated phosphorylation does not affect MTP8 protein stability or tonoplast localization, indicating that regulation occurs at the level of transport activity rather than protein abundance or targeting [18]. Mutations in either BRI1 or CNGC12 abolish CPK5-dependent phosphorylation of MTP8, placing the BRI1–CNGC12 module upstream of the CPK5–MTP8 cascade [24] (Figure 2, middle layer—pink).

#### 3.2.3. Excess Response II: CBL–CIPK-Mediated Suppression of MTP8

Following CPK-mediated activation, a delayed ‘brake’ mechanism engages to prevent over-sequestration. The tonoplast-localized Ca^2+^ sensors CBL2/3 recruit CIPK3/9/26 to phosphorylate MTP8 at a distinct site, Ser35. This phosphorylation suppresses MTP8 activity, as evidenced by the fact that phosphomimetic S35D fails to transport Mn in yeast, while non-phosphorylatable S35A causes excessive vacuolar Mn accumulation and impaired growth under moderate Mn supply [20]. Kinetic analyses reveal a temporal cascade: CPK-mediated activation (Ser31/32) peaks at 6 h of Mn exposure, whereas CBL–CIPK-mediated suppression (Ser35) peaks at 24 h (Figure 2, middle layer—pink). Thus, the same transporter is sequentially activated and then suppressed by distinct Ca^2+^ sensors—CPKs for rapid detoxification and CBL–CIPKs for fine-tuning—exemplifying the sophistication of Ca^2+^-mediated control.

#### 3.2.4. Excess Response III: The CBL1/9–CIPK23 Module Orchestrates Phosphorylation-Dependent Endocytosis of NRAMP1

In the presence of excess Mn, the high-affinity Mn transporter NRAMP1 is rapidly internalized from the plasma membrane into endosomal compartments, a process that is essential for limiting Mn uptake and preventing toxicity [34]. Time-course analyses revealed that NRAMP1 endocytosis begins within 30 min of Mn exposure and becomes pronounced by 6 h, coinciding with the activation kinetics of upstream kinases [22]. Endocytosis is triggered by phosphorylation of NRAMP1’s N-terminal cytosolic domain, with Ser20 identified as a critical residue. Non-phosphorylatable mutations stabilize NRAMP1 at the plasma membrane, whereas phosphomimetic mutations cause constitutive internalization [34] (Figure 2, middle layer—pink). The role of Ser22 remains debated, with some evidence suggesting cooperative function with Ser20, but this requires further clarification [22,34].

**Figure 3:**
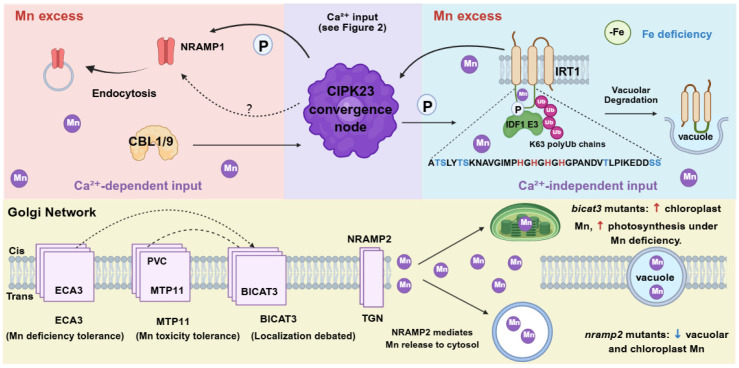
Network Integration–Signal Convergence and Inter-Organellar Mn Allocation. (**Top panel**) Proposed convergence on CIPK23 (working hypothesis). CIPK23 (purple) integrates Ca^2+^-dependent input (CBL1/9–CIPK23 → NRAMP1 endocytosis see Section 3.2.4) and Ca^2+^-independent input (IRT1 metal sensing → CIPK23 → IDF1-mediated degradation; see Section 3.4). Dashed arrows and ‘?’ indicate debated requirement of CIPK23 for NRAMP1 endocytosis (see Section 3.2.4). (**Bottom panel**) Golgi network and inter-organellar Mn allocation. Transporters coordinate Mn fluxes: ECA3 (Mn import under deficiency); MTP11 (toxicity tolerance; directly regulated by Ca^2+^-dependent CBL2/3–CIPK3/9/26; see Section 3.2.5); BICAT3 (Mn^2+^/Ca^2+^ transport, localization debated); and NRAMP2 (Mn release from TGN). Mutant names in lower case italics (e.g., *bicat3*, *nramp2*). Abbreviations: CIPK23, CBL-interacting protein kinase 23; CBL1/9, calcineurin B-like proteins; NRAMP1/2, natural resistance-associated macrophage proteins; IRT1, iron-regulated transporter 1; IDF1, IRT1 degradation factor 1; ECA3, ER-type Ca^2+^-ATPase 3; MTP11, metal tolerance protein 11; BICAT3, bivalent cation transporter 3; TGN, trans-Golgi network; PVC, prevacuolar compartment. Color coding: red, Ca^2+^-dependent; blue, Ca^2+^-independent; purple, convergence node (provisional); yellow, Golgi network. Arrows: dashed arrows, debated/hypothetical; solid arrows, established connections; red arrows, increase; blue arrows, decrease.

Several kinases have been implicated. CPK21/23 phosphorylates Ser20 in vitro, but *cpk21 cpk23* double mutants exhibit normal NRAMP1 internalization, indicating that they are not required for Mn-induced endocytosis [19]. In contrast, the CBL1/9–CIPK23 module plays a central role: CBL1/9 recruits CIPK23 to phosphorylate NRAMP1 at Ser20/22 (and also at Thr498 in the C-terminus), which is induced by excess Mn and peaks at 6 h [22]. Both *cipk23* and *cbl1 cbl9* mutants are hypersensitive to excess Mn and exhibit impaired NRAMP1 endocytosis [22]. However, the requirement of CIPK23 remains debated. While Zhang et al. [22] reported that NRAMP1 endocytosis is abolished in *cipk23* mutants, Kosuth et al. [30] found that endocytosis persists in a *cipk23* background, suggesting functional redundancy among kinases. Regardless of the kinase(s) involved, preventing NRAMP1 internalization via the phosphodead S20A mutation severely compromises plant tolerance to Mn excess [34] (Figure 3, top panel).

Upstream of this process, the BRI1–CNGC12 module generates the Mn-induced Ca^2+^ signal required for CBL1/9–CIPK23-mediated endocytosis, as mutations in BRI1 or CNGC12 impair NRAMP1 internalization [24]. Together, these findings establish phosphorylation-dependent endocytosis of NRAMP1 as a critical safeguard against Mn toxicity.

#### 3.2.5. Extension to MTP11: A Parallel Brake Target of CBL2/3–CIPK3/9/26

Very recently, the same CBL2/3–CIPK3/9/26 module has been shown to regulate MTP11, a Golgi/prevacuolar-localized Mn efflux transporter [21]. Under prolonged high Mn stress (36 h), this module phosphorylates MTP11 at Ser194/201, triggering its 26S proteasomal degradation and retaining it in the endomembrane system (Golgi/PVC), thereby reducing Mn exocytosis. Phospho-dead variants (S194A, S201A) confer enhanced Mn tolerance, whereas phospho-mimetic variants (S194D, S201D) cause hypersensitivity, mirroring the functional logic observed for MTP8. Notably, the peak of MTP11 phosphorylation (36 h) occurs later than that of MTP8 suppression (24 h), establishing a temporal hierarchy: early CPK-mediated activation of vacuolar sequestration (MTP8, 6 h), followed by CBL–CIPK-mediated suppression of vacuolar sequestration (MTP8, 24 h), and finally suppression of exocytic efflux (MTP11, 36 h; Figure 2, middle layer—pink). This multi-tiered brake system ensures that under sustained Mn stress, plants first sequester excess Mn into vacuoles, then gradually reduce both vacuolar uptake and apoplastic export to maintain cytosolic Mn within a physiological window. The discovery of MTP11 as a second target of the CBL2/3–CIPK3/9/26 module underscores the broader role of this Ca^2+^-dependent signaling hub in coordinating multiple transporters across different subcellular compartments.

### 3.3. A Parallel Ca^2+^-Independent Pathway: ROP6–MTP8

Recent studies have uncovered a Ca^2+^-independent route that also targets MTP8. Under Mn excess, the small GTPase ROP6 is converted to its active GTP-bound form, which then physically interacts with MTP8 [23]. This interaction enhances MTP8 localization to the tonoplast and increases its transport efficiency in *Arabidopsis* roots. The *rop6* mutant shows reduced vacuolar Mn accumulation and increased Mn sensitivity, whereas constitutively active ROP6 enhances tolerance. ROP6 activation does not require Ca^2+^ and operates in parallel with the Ca^2+^-dependent pathways, providing robustness to the detoxification system (Figure 2, middle layer—blue).

### 3.4. IRT1: A Paradigm of Metal-Sensing Transporter Regulation

Beyond the Ca^2+^-dependent mechanisms, the iron-regulated transporter IRT1 exemplifies an alternative paradigm—direct metal sensing by the transporter itself. IRT1 is the primary high-affinity Fe^2+^ transporter in *Arabidopsis* roots under iron deficiency, but it also transports Mn^2+^ as a secondary substrate [25] (Figure 1; middle panel). Under Mn excess, IRT1 acts as a transceptor: its histidine-rich intrinsically disordered loop binds Mn^2+^ with nanomolar affinity, recruiting CIPK23 kinase, which triggers IDF1-mediated K63-linked polyubiquitination and vacuolar degradation [25,26] (Figure 2, middle layer—blue). This Ca^2+^-independent pathway provides rapid feedback to limit further Mn uptake when cytosolic levels become excessive. A distinct Ca^2+^-dependent regulation of IRT1 occurs under iron deficiency: CPK21/23 phosphorylates IRT1 at Ser149, enhancing iron—not manganese—transport [10]. This pathway is not part of Mn homeostasis and is mentioned only to avoid confusion.

Notably, the same kinase CIPK23 that mediates IRT1 degradation also participates in NRAMP1 endocytosis (Section 3.2.4), raising the possibility that it may serve as a convergence point between Ca^2+^-dependent (NRAMP1) and Ca^2+^-independent (IRT1) pathways. However, given the debated requirement of CIPK23 for NRAMP1 endocytosis, this convergence model remains a working hypothesis (Figure 3, top panel).

The IRT1 pathway provides local, autonomous feedback based on a cytoplasmic metal load, curtailing Mn uptake when cytosolic concentrations rise. In contrast, Ca^2+^ signaling enables systemic coordination of multiple transporters (NRAMP1, MTP8, MTP11) in response to external Mn fluctuations. Together, these parallel mechanisms ensure that Mn uptake is precisely tuned to both cellular demand and environmental availability.

### 3.5. Scope and Limitations of Current Evidence

The studies summarized above firmly establish Ca^2+^-mediated regulation of three key Mn transporters—NRAMP1, MTP8 and MTP11—in *Arabidopsis*. For each, phosphorylation sites, upstream kinases, and physiological consequences have been validated by genetic and biochemical approaches. The recent identification of the BRI1–CNGC12 module provides the first molecular link between Mn perception and downstream Ca^2+^ signals, completing a signaling cascade from sensor to effector [24].

However, it is critical to recognize several limitations that temper the generality of this framework. First, and most importantly, all current mechanistic data derive from *Arabidopsis thaliana*; whether the same regulatory modules operate in crops, hyperaccumulators, or other plant species remains completely untested. Second, even within *Arabidopsis*, the direct evidence for Ca^2+^-dependent regulation is restricted to NRAMP1, MTP8, and MTP11; other Mn transporters (NRAMP3/4, CAX2, other MTPs) have not been examined for Ca^2+^-dependent phosphorylation. Third, the regulatory landscape includes both Ca^2+^-dependent and Ca^2+^-independent pathways (IRT1, ROP6), which converge only partially. Thus, the designation of Ca^2+^ signaling as a ‘key integrator’ applies strictly to the *Arabidopsis* NRAMP1/MTP8/MTP11 system; generalization to other transporters, species, or broader Mn homeostasis processes is a hypothesis to be tested, not an established fact.

These considerations highlight the need for a tentative hierarchical framework that distinguishes between processes with different levels of evidentiary support, while acknowledging that many assignments may be revised as new data emerge. The following section therefore organizes the current knowledge into three categories: (i) directly Ca^2+^-regulated processes, for which transporter-level evidence exists (Mn uptake via NRAMP1, vacuolar sequestration via MTP8); (ii) indirectly influenced processes, which depend on these transporters (tissue-level Mn distribution, remobilization); and (iii) candidate processes, where Ca^2+^ involvement is plausible but unproven (root architectural remodeling, organic acid exudation, metabolic reprogramming). This framework acknowledges current knowledge while providing testable hypotheses for future studies.

## 4. Integrating Ca^2+^ Signaling with Downstream Adaptive Strategies

Building on the hierarchical framework outlined in Section 3.5, this section examines how Ca^2+^-dependent regulation of core transporters interfaces with the broader repertoire of plant-adaptive responses to Mn stress. We first discuss processes for which direct Ca^2+^ regulation has been demonstrated, then consider those indirectly influenced by these transporters, and finally evaluate candidate processes in which a connection to Ca^2+^ signaling remains hypothetical. This structure is designed to clearly distinguish between established knowledge and areas requiring further investigation.

### 4.1. Directly Ca^2+^-Regulated Processes

Mn uptake via NRAMP1. The CPK21/23–NRAMP1 phosphorylation module directly enhances Mn influx into the plasma membrane under deficiency conditions [19]. Conversely, during excess, CBL1/9–CIPK23-mediated endocytosis removes NRAMP1 from the surface, reducing its uptake [22]. These reciprocal controls ensure that the net Mn influx matches external availability.

Vacuolar sequestration via MTP8. The CPK5–MTP8 activation module [18] and the CBL–CIPK–MTP8 suppression module [20] together provide dynamic control over vacuolar Mn storage. Kinetic analyses revealed that CPK5-mediated activation peaks early (6 h) to sequester excess Mn, while CBL–CIPK-mediated suppression peaks later (24 h) to prevent over-sequestration, fine-tuning Mn homeostasis under prolonged stress [20]. The ROP6 pathway adds a parallel input that enhances MTP8 vacuolar localization [23].

Suppression of Mn efflux via MTP11. The Golgi/prevacuolar-localized Mn efflux transporter MTP11 is also directly regulated by Ca^2+^ signals. Under prolonged high Mn stress, the CBL2/3–CIPK3/9/26 module phosphorylates MTP11 at Ser194/201, triggering its 26S proteasomal degradation and retaining it in the endomembrane system, thereby reducing Mn exocytosis [21] (see Section 3.2.5 for detailed mechanism). This late-phase brake (peak at 36 h) complements the MTP8 brake (peak at 24 h), together forming a multi-tiered temporal cascade that sequentially suppresses vacuolar sequestration and then exocytic efflux to maintain cytosolic Mn homeostasis under sustained stress.

### 4.2. Indirectly Influenced Processes

Tissue-level Mn distribution. NRAMP1 and MTP8 activities influence the amount of Mn that is available for long-distance transport. In *nramp1* mutants, shoot Mn accumulation is reduced under deficiency [19]. In rice, OsNRAMP3 functions as a node-based switch: under sufficient Mn supply, it loads Mn into xylem transfer cells for distribution to young leaves; under excess, OsNRAMP3 is degraded, allowing Mn to follow the transpiration stream to old leaves [13]. However, it remains unclear whether Ca^2+^ signaling triggers OsNRAMP3 degradation. Additionally, OsNRAMP5 is localized in the xylem parenchyma cells of rice leaf sheaths to unload Mn and restrict its translocation to the leaf blade, revealing a second function beyond root uptake [35].

Mn remobilization. NRAMP3 and NRAMP4 export Mn from vacuoles during deficiency [14]. Although these transporters have not been shown to be directly phosphorylated by Ca^2+^-dependent kinases, their activity could be influenced by changes in cytoplasmic Mn concentrations that result from Ca^2+^-regulated influx and sequestration. In *Arabidopsis*, AtNRAMP2 localizes to the trans-Golgi network/early endosomes and is required for Mn supply to chloroplasts; genetic analyses indicate that AtNRAMP2 and AtNRAMP3/4 operate successively, but how their activities are coordinated remains unclear [3].

Cell wall immobilization. The cell wall, enriched with negatively charged carboxyl and hydroxyl groups on pectins and hemicellulose, serves as the first barrier to Mn^2+^ entry [36]. In sugarcane, the Mn-tolerant genotype YC96-66 retains ~93% of leaf Mn in the pectic fraction of the cell wall, which is associated with increased pectin synthesis and demethylesterification [37]. Recent studies on mulberry have revealed that cellulose, hemicellulose, and lignin contents are significantly altered under Mn deficiency and toxicity, suggesting active cell wall remodeling in response to Mn stress [38]. Whether Ca^2+^ signaling directly regulates these modifications remains unknown; however, Ca^2+^ is known to influence pectin cross-linking and cell wall mechanics.

Golgi apparatus and other secretory pathway transporters (with Ca^2+^ regulation not yet established). The Golgi apparatus plays a central role in Mn-dependent glycosylation reactions, and multiple transporters contribute to Golgi Mn homeostasis. ECA3, a P_2_A-type ATPase localized to the trans-Golgi, is critical for maintaining tolerance under manganese-limiting conditions [39,40]. MTP10, another trans-Golgi-localized MTP, contributes to Mn detoxification only when both MTP8 and MTP11 are non-functional [40]. The bivalent cation transporter BICAT3 functions as a Mn^2+^/Ca^2+^ transporter in the Golgi apparatus, although its precise subcellular localization remains debated [41]. Under Mn deficiency, bicat3 mutants exhibit severe growth defects, altered cell wall composition, and impaired pollen tube growth due to defective pectin deposition, yet paradoxically show improved photosynthetic efficiency and higher chloroplast Mn content, indicating that BICAT3 influences inter-organellar Mn allocation [18,41]. In addition to Mn import, efflux from the Golgi is equally critical. The TGN-localized transporter NRAMP2 mediates Mn release into the cytosol, supplying various organelles and Mn-dependent enzymes [42,43]. Loss of NRAMP2 reduces vacuolar and chloroplast Mn content and increases sensitivity to Mn deficiency, underscoring the role of Golgi-derived Mn in cellular distribution [3,42]. The central role of the Golgi apparatus in Mn homeostasis appears to be evolutionarily conserved, as yeast and mammalian homologs similarly regulate Mn-dependent glycosylation [3,44]. For these Golgi-localized transporters (ECA3, MTP10, BICAT3, NRAMP2), whether they are subject to Ca^2+^-dependent regulation remains largely unexplored. However, given the presence of Ca^2+^-permeable channels and Ca^2+^-binding proteins in the Golgi membrane, this possibility warrants future investigation. (Note: MTP11, also Golgi-localized, is directly regulated by Ca^2+^ signaling and is discussed in Section 4.1; Figure 3, bottom panel).

The processes described above—tissue distribution, remobilization, and cell wall binding—are influenced by the activities of Ca^2+^-regulated transporters but have not themselves been shown to be directly Ca^2+^-dependent. We now turn to candidate processes for which a connection to Ca^2+^ signaling is plausible but entirely unproven.

### 4.3. Candidate Processes Awaiting Validation

Root architectural remodeling. Mn deficiency alters root hair development and lateral root density [6,12]. In *Arabidopsis*, delayed endodermal suberization under Mn, Fe, and Zn deficiency depends on ethylene signaling and likely serves to increase the absorptive area [3]. In barley, the strength of Mn deficiency determines endodermal suberization patterns—increasing uptake into the xylem under mild deficiency but decreasing leakage under severe deficiency [45]. Ca^2+^ is a known regulator of root development under other stresses, but whether Ca^2+^ signals directly mediate Mn-driven root remodeling remains unknown. Phenotyping of Ca^2+^ signaling mutants under Mn deficiency could address this gap.

Organic acid exudation. Organic acid secretion is a common strategy for metal detoxification. In *Stylosanthes guianensis*, Mn stress induces malate dehydrogenase SgMDH1, which increases malate synthesis and exudation; secreted malate forms stable Mn-malate complexes, reducing free Mn^2+^ in the rhizosphere [46]. Malate exudation can be triggered by Al^3+^ via a Ca^2+^-dependent pathway involving the ALMT1 transporter [47], but whether a similar mechanism operates under Mn deficiency remains unknown.

Metabolic reprogramming. Transcriptomic and metabolomic studies have revealed extensive reprogramming under Mn stress, with common themes including activation of the phenylpropanoid pathway (leading to accumulation of ROS-scavenging phenolics and flavonoids), as well as alterations in α-linolenic acid metabolism and carbohydrate metabolism [38,48,49,50]. While Ca^2+^ signals can influence these pathways through CPK-mediated transcription factor phosphorylation, direct connections to Mn-specific responses have not been established.

Antioxidant system activation. Both enzymatic (SOD, CAT, POD and APX) and non-enzymatic (ascorbate, glutathione and flavonoids) antioxidants are induced under Mn stress [16,51]. In soybean, SOD, CAT, APX, and POD activities rapidly increase with Mn concentration [51]. In the Mn hyperaccumulator *Polygonum lapathifolium*, high SOD, CAT, and APX activities are maintained in both the roots and stems [16]. The ascorbate–glutathione cycle effectively eliminates excess ROS in *Stylosanthes* under Mn toxicity [16]. Comparative studies between the Mn hyperaccumulator *Phytolacca americana* and tobacco have shown that *P. americana* maintains higher SOD and continuously increasing CAT activity under Mn stress, whereas tobacco CAT declines sharply, suggesting that sustained antioxidant capacity is a key hyperaccumulator trait [38].

The candidate processes discussed above share a common feature: they have been documented to occur under Mn stress, but their direct regulation by Ca^2+^ signals remains hypothetical. Before concluding, we examine how the core Ca^2+^-dependent mechanisms may vary across plant species, potentially explaining differences in Mn tolerance.

### 4.4. Species-Specific Variations

Crop cultivars. Major crop species exhibit marked differences in Mn tolerance thresholds. Rice (*Oryza sativa*) is relatively Mn-tolerant, with toxicity symptoms typically appearing only at tissue concentrations far exceeding those that are phytotoxic to sensitive species. In contrast, maize (*Zea mays*) is highly susceptible and exhibits growth inhibition at much lower tissue Mn concentrations [7,11]. Wheat (*Triticum aestivum*) displays intermediate tolerance, with a range of genotypic variation spanning concentrations between the thresholds of sensitive and tolerant species [6]. Genotypic differences in Mn tolerance are often associated with the differential expression or activity of transporters and chelators. In sugarcane, the tolerant genotype YC96-66 sequesters excess Mn in cell wall pectin, whereas the sensitive genotype YC58-21 accumulates Mn in the cytoplasm, leading to toxicity [37]. These contrasting strategies likely reflect differences in the regulation or activity of Ca^2+^-signaling modules; however, direct evidence is lacking. With the availability of high-quality genome sequences and GWAS resources for major crops, future studies may identify natural allelic variation in signaling components (e.g., *CPK*, *CBL* and *CIPK*) that underlie the diversity of Mn tolerance.

Hyperaccumulators. Approximately 24 Mn hyperaccumulator species have been documented worldwide, with foliar Mn concentrations exceeding 10,000 mg kg^−1^DW without toxicity symptoms [52]. Notable examples include *Celosia argentea* (up to 17,961 mg kg^−1^), *Phytolacca americana*, and *Polygonum perfoliatum* [52,53]. Hyperaccumulators employ enhanced vacuolar sequestration, cell wall binding, and organic acid chelation mechanisms. In *P. americana*, oxalate is the dominant chelator and forms MnC_2_O_4_·3H_2_O crystals [53]. In *Austromyrtus bidwillii*, succinic acid predominates [54]. Synchrotron μ-XRF mapping has revealed that Mn is preferentially localized in the leaf epidermis, stem cortex, and root cortex/phloem in the hyperaccumulator *Grevillea meisneri*, with strong Mn–Ca co-localization suggesting coordinated sequestration [55]. These tissue-level distribution patterns minimize interference with the photosynthetic mesophyll cells. The molecular basis of hyperaccumulation remains largely unknown. Whether these species have evolved modified Ca^2+^ signaling modules, altered phosphorylation sites in transporters, or enhanced expression of chelator biosynthetic genes is a key question for future comparative studies.

Evolutionary conservation of MTP8 regulatory phosphorylation sites. Comparative sequence analysis of MTP8 homologs across 14 plant species reveals differential conservation of the activating and suppressive phosphorylation sites, providing molecular insight into the species-specific strategies discussed above. The activating Ser31/32 residues targeted by CPKs are highly conserved in both dicots and monocots, suggesting that CPK-mediated activation of MTP8 may represent a relatively conserved mechanism across plant species [20]. By contrast, the suppressing Ser35 residue targeted by CBL–CIPK complexes is conserved only in dicots; in monocots, this residue is replaced by a glycine (G), while the neighboring residue is replaced by a potentially phospho-mimetic glutamate (E), and a conserved Ser39 residue appears in all analyzed monocots [20]. However, these sequence-based predictions require direct experimental validation in monocot species; functional equivalence of the alternative residues has not been demonstrated. This sequence and p-site pattern suggest that monocots rely on an MTP8 deactivation mechanism that differs from that of dicots.

## 5. Future Directions: From Mechanism to Application

### 5.1. Unresolved Questions

The framework developed above raises several fundamental questions that define the roadmap for future research. First, what is the complete set of upstream Mn sensors? The identification of CNGC12 as a key channel for Mn-induced Ca^2+^ signals represents a major advance, but how CNGC12 itself perceives Mn status remains unknown. Does it directly bind Mn^2+^, or is it activated by an intermediate Mn-binding protein? The BRI1–CNGC12 interaction adds another layer: brassinosteroid signaling is now linked to Mn stress adaptation. However, whether BR levels change under Mn stress and how BRI1 kinase activity is rapidly activated by Mn requires investigation [24].

Second, how are different Ca^2+^ signatures (oscillations vs. transients) decoded to activate specific kinase modules? CPK21/23 may require sustained Ca^2+^ for full activation owing to slow autophosphorylation, whereas CPK5 may respond to rapid spikes. The subcellular localization of different CBL–CIPK complexes (tonoplast vs. plasma membrane) may also contribute to signal specificity in plants. Spatiotemporally resolved Ca^2+^ imaging combined with optogenetic control of Ca^2+^ patterns could test these hypotheses.

Third, how do the parallel pathways—Ca^2+^-dependent (NRAMP1/MTP8) and Ca^2+^-independent (IRT1, ROP6)—coordinate to fine-tune Mn homeostasis? The discovery that CIPK23 serves as a convergence node for both IRT1 degradation and NRAMP1 endocytosis raises the question of how this kinase is differentially regulated by distinct upstream signals. Does the same CIPK23 pool respond to both cytoplasmic metal loads (via IRT1) and Ca^2+^ signals (via CBL1/9)? Are there spatially segregated pools? Understanding this integration is crucial for predicting plant responses to combined stresses (e.g., Fe deficiency plus Mn excess).

Fourth, to what extent are the regulatory mechanisms conserved across species? Recent reviews emphasize that “the emergent diversity of Mn handling beyond the *Arabidopsis* model” is a key frontier [3]. Natural variation in *CPK*, *CBL*, and *CIPK* genes among crop cultivars [56] could be exploited for breeding. Genome-wide association studies of Mn tolerance traits may identify alleles in these signaling components. The recent discovery that phosphorylation of STNP residues in SaNRAMP5 governs Cd/Mn selectivity and plasma membrane localization [57] suggests that engineering phosphorylation sites could alter metal specificity without compromising essential Mn uptake.

Fifth, what is the interplay between the BRI1–CNGC12 hormone signaling module and the IRT1 metal-sensing pathway? Both ultimately influence CIPK23 activity—BR via Ca^2+^ and CNGC12, and IRT1 via direct metal binding—but whether cross-talk exists is unknown.

Sixth, do BICAT family transporters, which mediate Mn and Ca^2+^ fluxes across chloroplast and Golgi membranes, interact with the Ca^2+^ signaling network? Their dual substrate specificity and the existence of Ca^2+^-dependent processes in these organelles raise the possibility of cross-talk between Mn and Ca^2+^ homeostasis at the organellar level [3].

### 5.2. Translational Opportunities

The detailed knowledge of phosphorylation sites offers precision targets for genetic improvement. Constitutively active variants of MTP8 (S31/32D) could enhance Mn sequestration in sensitive crops grown on acidic soils, while phosphodead NRAMP1 (S20/22A) might maintain Mn uptake under excess for phytoremediation applications. The demonstration that mutating STNP residues in SaNRAMP5 reduces Cd uptake without affecting Mn uptake [57] provides a proof-of-concept for engineering low-Cd crops without yield penalties. CRISPR base editing enables such codon-specific changes without introducing transgenes, making these approaches feasible for elite cultivars.

Exogenous interventions can also be reinterpreted using the Ca^2+^ lens. Silicon application, which mitigates Mn toxicity [58,59], may alter the cell wall properties that affect mechanosensitive Ca^2+^ channels. Mn oxide nanoparticles [60] might slowly release Mn^2+^, generating sustained low-amplitude Ca^2+^ signals that prime adaptive responses. The finding that brassinolide treatment enhances Mn-induced Ca^2+^ signaling and Mn tolerance [24] suggests that exogenous BR application could be explored as an agronomic strategy for managing Mn toxicity. Microbial inoculants that oxidize Mn^2+^ in the rhizosphere [61] reduce free Mn^2+^ levels and could attenuate excess-induced Ca^2+^ transients. These hypotheses are testable and could guide the rational design of agronomic practices.

## 6. Conclusions

The past five years have transformed our understanding of Mn homeostasis in *Arabidopsis* from a collection of isolated observations to an emerging framework organized around Ca^2+^ signaling. Direct evidence now demonstrates that Ca^2+^-dependent phosphorylation dynamically regulates the activity and turnover of key Mn transporters—NRAMP1, MTP8 and MTP11—in *Arabidopsis*. The recent discovery that the CBL2/3–CIPK3/9/26 module dually targets MTP8 (vacuolar sequestration, peak 24 h) and MTP11 (exocytic efflux, peak 36 h) in a temporally staggered manner [21] illustrates how Ca^2+^ signaling coordinates multiple transporters across different subcellular compartments to achieve integrated Mn homeostasis. The identification of the BRI1–CNGC12 module as the upstream generator of Mn-induced Ca^2+^ signals [24] provides the first molecular link from Mn perception to downstream regulation. However, the framework is explicitly presented as an *Arabidopsis*-centered working model, not a universally established principle. Several critical limitations remain: (i) all direct mechanistic evidence comes from *Arabidopsis*; validation in crops and hyperaccumulators is urgently needed. (ii) The requirement of CIPK23 for NRAMP1 endocytosis remains debated [22,30]. (iii) Many adaptive strategies—root architectural remodeling, cell wall modification, metabolic reprogramming—lack direct evidence of Ca^2+^ involvement. By clearly distinguishing established, indirect, and hypothetical connections, this review provides a useful framework to guide future research, with the understanding that extension to other species and broader physiological contexts requires experimental validation.

## Data Availability

All data discussed are available in the cited references. No new data were generated for this study.
